# Enzyme-Enzyme Interactions in Monolignol Biosynthesis

**DOI:** 10.3389/fpls.2018.01942

**Published:** 2019-01-11

**Authors:** Jack P. Wang, Baoguang Liu, Yi Sun, Vincent L. Chiang, Ronald R. Sederoff

**Affiliations:** ^1^ Forest Biotechnology Group, Department of Forestry and Environmental Resources, North Carolina State University, Raleigh, NC, United States; ^2^ State Key Laboratory of Tree Genetics and Breeding, Northeast Forestry University, Harbin, China; ^3^ Department of Forestry, Beihua University, Jilin, China

**Keywords:** monolignol biosynthesis, protein-protein interaction, lignin, enzyme kinetics, BiFC, co-immunoprecipitation

## Abstract

The enzymes that comprise the monolignol biosynthetic pathway have been studied intensively for more than half a century. A major interest has been the role of pathway in the biosynthesis of lignin and the role of lignin in the formation of wood. The pathway has been typically conceived as linear steps that convert phenylalanine into three major monolignols or as a network of enzymes in a metabolic grid. Potential interactions of enzymes have been investigated to test models of metabolic channeling or for higher order interactions. Evidence for enzymatic or physical interactions has been fragmentary and limited to a few enzymes studied in different species. Only recently the entire pathway has been studied comprehensively in any single plant species. Support for interactions comes from new studies of enzyme activity, co-immunoprecipitation, chemical crosslinking, bimolecular fluorescence complementation, yeast 2-hybrid functional screening, and cell type–specific gene expression based on light amplification by stimulated emission of radiation capture microdissection. The most extensive experiments have been done on differentiating xylem of *Populus trichocarpa*, where genomic, biochemical, chemical, and cellular experiments have been carried out. Interactions affect the rate, direction, and specificity of both 3 and 4-hydroxylation in the monolignol biosynthetic pathway. Three monolignol P450 mono-oxygenases form heterodimeric and heterotetrameric protein complexes that activate specific hydroxylation of cinnamic acid derivatives. Other interactions include regulatory kinetic control of 4-coumarate CoA ligases through subunit specificity and interactions between a cinnamyl alcohol dehydrogenase and a cinnamoyl-CoA reductase. Monolignol enzyme interactions with other pathway proteins have been associated with biotic and abiotic stress response. Evidence challenging or supporting metabolic channeling in this pathway will be discussed.

## Introduction

Metabolic pathways are typically conceived as sequences of enzymatic events that are linear, branched, or sometimes circular, that is, how they are described in charts and textbooks. It is a standard procedure in biochemistry to study enzyme reactions in highly dilute aqueous solutions, that is, what is needed to isolate and characterize specific enzymes. As a result, the roles of structure and compartmentation may not be adequately considered. We often assume that each enzyme acts independently, in part because we must purify each enzyme to learn its essential properties, unconfounded by activity of other enzymes. In biochemical reality, enzymes and pathways occur in three-dimensional space and are likely to be associated with other proteins, polymers, membranes, and intracellular structures.

Much of our genetic theory of selection is based on concepts of genes acting independently, at least in part because it provides a computational simplification, although interactions of genes and proteins abound. Genomics provides a new platform for the investigation of molecular interactions and structure because all members of gene and protein families can now be identified and characterized, so that assays for molecular interactions can now be investigated in a far more comprehensive way. We can investigate the role of structure for pathways, beginning from the investigation of protein-protein interactions using new tools to detect genome wide enzymatic and physical interactions.

**Figure 1:**
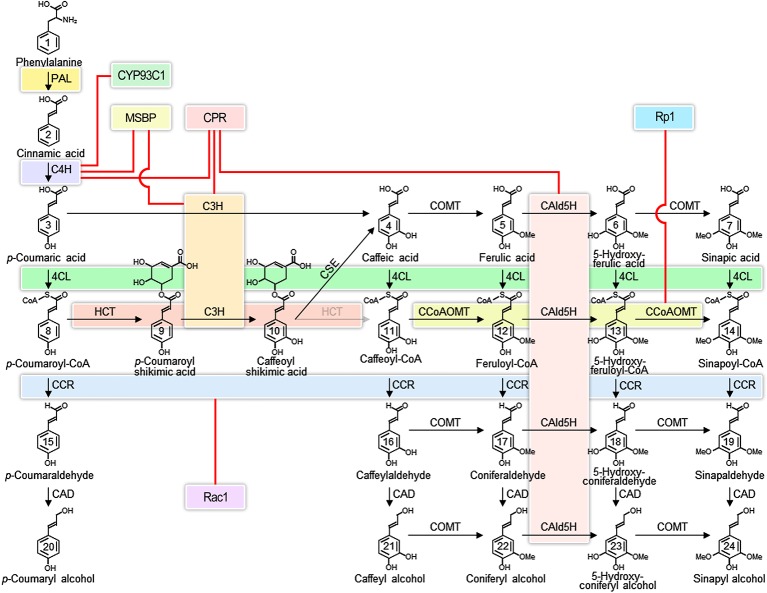
Interacting enzyme in the monolignol biosynthetic pathway. Colored boxes represent the interacting enzyme families. Red lines represent interactions indicated in the literature that involve proteins outside of the core monolignol pathway. Abbreviations: phenylalanine ammonia-lyase (PAL); cinnamate 4-hydroxylase (C4H); coumarate 3-hydroxylase (C3H); 4-coumarate: coenzyme A ligase (4CL); hydroxycinnamoyl-CoA shikimate hydroxycinnamoyl transferase (HCT); caffeoyl shikimate esterase (CSE); caffeoyl coenzyme A O-methyltransferase (CCoAOMT); cinnamoyl-CoA reductase (CCR); coniferaldehyde 5-hydroxylase (CAld5H); 5-hydroxyconiferaldehyde O-methyltransferase (COMT); cinnamyl alcohol dehydrogenase (CAD); membrane steroid-binding protein (MSBP); cytochrome P450 reductase (CPR); isoflavone synthase (CYP93C1); plant disease resistance gene 1 (Rp1); Rac family small GTPase 1 (Rac1).

The lignin biosynthetic pathway in vascular plants (Figure 1) has been intensively studied for the greater part of a century (Payen, 1839) because of its fundamental role in the formation of the plant secondary cell wall (Sarkanen and Ludwig, 1971; Harada and Côté, 1985; Boerjan et al., 2003; Weng and Chapple, 2010) and for its practical implications in wood formation (Sarkanen and Ludwig, 1971; Hu et al., 1999; Chiang, 2002; Rastogi and Dwivedi, 2006; Chen and Dixon, 2007; Vanholme et al., 2008; Novaes et al., 2010; Wang et al., 2014; Wang et al., 2018). The lignin biosynthetic pathway begins in the cytoplasm by deaminating phenylalanine and proceeds stepwise through the modification of the phenyl ring and the reduction of the propanoid side chain to produce the monolignol precursors, which are translocated to the apoplast to form the lignin polymer (Figure 1) (Higuchi, 1985; Boerjan et al., 2003; Wang et al., 2014). While each step of the pathway has reversible reactions, which can be described by Michaelis-Menten kinetics (Wang et al., 2014), the formation of the lignin polymer by oxidative polymerization is not reversible.

The lignin pathway has been variously described as linear, branched, or metabolic grid. The pathway is different in different taxa, related to the distinction of hardwoods (angiosperms) that make a mixed polymer from guaiacyl and syringyl monolignols, compared to softwoods (gymnosperms) that make a purely guaiacyl lignin (Mottiar et al., 2016). Different cell types have different lignin compositions (Fergus and Goring, 1970a,b; Zhou et al., 2011; Shi et al., 2017). Vessel cells in poplar xylem have a guaiacyl rich lignin, while adjacent fiber cells have a syringyl rich lignin content. Studies typically have not been comprehensive in which all the enzymes in the pathway have not been studied in the same cell types, and comparisons across species have been both informative and confusing. The full extent of variation in lignin at the cell, tissue, and species level has not yet been determined.

Our previous studies have shown that unexpected and dramatic effects on the metabolic flux through the monolignol pathway have been the result of protein-protein interactions (Chen et al., 2011, 2014; Naik et al., 2018; Yan et al., 2018). These interactions can affect the extent and direction of flux. The purpose of this short review is to describe the interactions that have been found to date, to guide the discovery of new interactions.

### Early Concepts and Evidence for Interactions

The aqueous environment within cells and the concentration of proteins in typical biochemical reactions are quite different. Protein concentrations in cells are very high, and the cellular environment in the *in vitro* reactions is dilute by comparison (Mendes et al., 1995). In theory, compartmentation would increase the concentration of metabolites and proteins leading to higher efficiencies (Ralston and Yu, 2006). In addition to compartmentation, molecular organization of the enzymes could lead to greater efficiencies by direct physical interaction of enzymes or by organizing structures bringing metabolites into greater proximity. “Supramolecular complexes of sequential metabolic enzymes and cellular structural elements” called metabolons have been proposed (Kuzin, 1970; Srere, 1985, 1987).

In 1974, Stafford presumed the existence of multienzyme complexes for phenylpropanoid metabolism because of the diversity of secondary products in the same cells and the need for a mechanism that regulated the complex series of biosynthetic pathways (Stafford, 1974). Support for multienzyme complexes was observed as high molecular weight aggregates, differences in utilization of endogenous versus exogenous origin of metabolites, and existence of multiple isoforms of enzymes. None of this, however, was unequivocal direct evidence for multienzyme complexes (Stafford, 1974). Early evidence for interactions as membrane bound enzyme complexes in monolignol biosynthesis came from studies of the formation of *p*-coumaric acid from phenylalanine in potato slices (Czichi and Kindl, 1975). Cinnamic acid added to a soluble reaction mixture was a less effective substrate for cinnamate 4-hydroxylase (C4H) than cinnamic acid formed by the phenylalanine ammonia-lyase (PAL) reaction (Czichi and Kindl, 1975, 1977). This observation indicated that endogenously formed cinnamic acid may only be partially equilibrated with exogenous cinnamic acid and that the interaction is dependent on microsomal integrity. PAL and C4H were subsequently proposed to interact and channel substrates for increased catalytic efficiency and regulation of flux (Rasmussen and Dixon, 1999; Achnine et al., 2004; Ralston and Yu, 2006). The concept of “metabolic channeling” refers to the transfer of a metabolite as a product of one enzyme to its interacting enzyme as substrate, without entering a metabolic pool (Winkel, 2004; Jorgensen et al., 2005). However, reconstituted PAL and C4H in yeast revealed that metabolic channeling is not required for the conversion of phenylalanine to *p*-coumaric acid (Ro and Douglas, 2004). Measurements of relative abundance of the 24 monolignol precursors also did not support the hypothesis of metabolic channels in this pathway (Chen et al., 2011). The intriguing hypothesis of channeling in monolignol biosynthesis remains to be substantiated.

Proteins in the endoplasmic reticulum (ER) are thought to act as anchors for multi-protein complexes (Ralston and Yu, 2006). Candidates for protein anchors in the phenylpropanoid pathway are the cytochrome P450s (Durst and Benveniste, 1993). P450s are the largest family of enzymes in plants. Enzymes catalyzing three of the oxidative steps of the monolignol biosynthetic pathway are cytochrome P450s. They are C4H, coumarate 3-hydroxylase (C3H), and ferulate 5-hydroxylase (F5H) also known as coniferaldehyde 5-hydroxylase (CAld5H) (Figure 1). C4H and C3H are early steps in the monolignol pathway, common to much of phenylpropanoid metabolism (Vogt, 2010), while CAld5H acts late at lignin-specific steps (Osakabe et al., 1999). These P450s insert an oxygen into hydrophobic regions of proteins and as a result increase reactivity and solubility. All cytochrome P450s are mono-oxygenases that split molecular oxygen through NADPH-cytochrome P450 reductases (CPRs), which need to be tightly coupled to the P450 (Omura, 2010). A hydrophobic membrane-spanning domain anchors the CPR to the ER. Evidence for a direct interaction of CPRs with P450s has been provided using atomic force microscopy and reconstituted phospholipid bilayer disks (Bayburt et al., 1998; Bayburt and Sligar, 2002). Surface residues of both the CPR and the P450 are thought to contribute to the CPR-P450 interaction. The number of P450s in plants far exceeds the number of CPRs therefore that any given CPR might couple with many different P450s, involved with other pathways (Ralston and Yu, 2006). Such interactions are suggested to be dynamic and transient because of the disparity in abundance.

### Direct Physical Interaction of Ptr4CL3 and Ptr4CL5 in *Populus trichocarpa*

Genome sequence analysis identified 17 putative genes in *P. trichocarpa* with similarity to known 4CL encoding genes (Shi et al., 2010). Of the 17, only two (*PtrPtr4CL3* and *PtrPtr4CL5*) encode functional enzymes for CoA ligation in stem differentiating xylem (Chen et al., 2013).

A simple experiment indicated that the activity of a mixture of the Ptr4CL3 and Ptr4CL5 isoforms was not additive (Chen et al., 2014). Further experiments demonstrated that the isoforms interact to form a tetramer, *in vitro* and *in vivo*, which appears to have a regulatory role (Chen et al., 2014). Laser microdissection was used to collect different cell types to determine that both isoforms of 4CL are present in the same cells. Most of the transcripts in differentiating xylem are in fiber cells (Chen et al., 2014; Shi et al., 2017; Wang et al., 2018). Isolated fiber cells obtained by microdissection showed that transcripts from both 4CL genes are co-expressed and abundant. Bimolecular fluorescence complementation (BiFC) was carried out using complementing fragments of yellow fluorescence protein (YFP) co-transfected into *P. trichocarpa* protoplasts from differentiating xylem (Chen et al., 2014; Lin et al., 2014). Strong complementation was observed, indicating close spatial proximity (6–10 nm, Fan et al., 2008) between Ptr4CL3 and Ptr4CL5 in the protoplasts.

Further evidence of an interaction between Ptr4CL3 and Ptr4CL5 has been obtained from chemical crosslinking using dithiobis (succinimidyl propionate) (DSP), which makes crosslinks equivalent to 8 carbon linkages, about 12 angstroms (Lomant and Fairbanks, 1976). A crosslinked mixture of Ptr4CL3 and Ptr4CL5 produced a band detected on SDS-PAGE greater than 200 kDa, consistent with a heterotetramer (Chen et al., 2014). Co-immunoprecipitation (Co-IP) also supported the existence of a protein complex involving Ptr4CL3 and Ptr4CL5 (Chen et al., 2014). Antibody prepared against either Ptr4CL3 or Ptr4CL5 was able to co-precipitate both Ptr4CL3 and Ptr4CL5 from extracts of differentiating xylem. Therefore, the complex could be formed *in vitro* and *in vivo*. The stoichiometry of the complex was calculated to be a Ptr4CL3: Ptr4CL5 ratio of 3:1 based on a size estimate of ~240 kDa by SDS-PAGE (Chen et al., 2014). The size of the monomer is ~60 kDa. A ratio of molecular mass of the complex from crosslinking, purification, and protein cleavage-isotope dilution mass spectrometry was 2.69 to 1 of Ptr4CL3 to Ptr4CL5, supporting the conclusion of a hetero-tetramer with three subunits of Ptr4CL3 and one of Ptr4CL5. A mathematical model was developed that predicts the metabolic flux for mixtures of the two enzymes, in the presence of multiple substrates, incorporating the activity of the monomers and tetramers including competitive inhibition, uncompetitive inhibition, and self-inhibition (Chen et al., 2013, 2014). The model suggests that Ptr4CL3-Ptr4CL5 complex improves the homeostatic properties of the pathway, increasing the stability of the pathway by 22% (Naik et al., 2018).

### Membrane Protein Complexes Catalyze 4- and 3-Hydroxylation of Cinnamic Acids in *P. trichocarpa*

As discussed earlier, the entry steps to the pathway for monolignol biosynthesis were hypothesized to involve metabolic channeling (Rasmussen and Dixon, 1999; Achnine et al., 2004; Ralston and Yu, 2006). Genomic sequence and transcriptome analysis indicate the involvement of multiple P450 proteins in these steps in *P. trichocarpa* (Shi et al., 2010). Two paralogs of C4H, designated PtrC4H1 and PtrC4H2, are abundantly and specifically expressed in fiber and vessel cells of stem differentiating xylem (Chen et al., 2011; Shi et al., 2017; Wang et al., 2018). One gene encodes the activity of C3H (PtrC3H3). All three are resident ER proteins (Chen et al., 2011).

Co-expression of some combinations of the three hydroxylases had increased activities (Chen et al., 2011) compared to individual enzymes in a yeast system (Urban et al., 1994). Co-expression of PtrC4H1 and PtrC3H3 showed up to 40-fold increases in *V*_max_ of 4-hydroxylase activity compared to the individual proteins, while PtrC4H2 and PtrC3H3 showed nearly a 100-fold increase in 4-hydroxylase activity. The combination of the three enzymes had a lower *K*_m_ with cinnamic acid and more than a 100-fold increase in catalytic efficiency (*V*_max_/*K*_m_) compared to the two C4H isoforms (Chen et al., 2011). Co-expression of PtrC4H1 and PtrC3H3 showed nearly a 500-fold increase in catalytic efficiency for 3-hydroxylation of *p*-coumaroyl shikimic into caffeoyl shikimic acid. Co-expression of all three hydroxylases, the increase was over 6,500-fold. Taken together with the activities in extracts of stem differentiating xylem, the results support two hydroxylation pathways, one for the conversion of *p*-coumaric to caffeic acid and the other for the conversion of *p*-coumaroyl shikimic acid to caffeoyl shikimic acid. These results suggest that when co-expressed in the same membrane system, the hydroxylases interact through protein-protein interactions to modulate enzyme activity and metabolic flux (Chen et al., 2011). This inference was further supported by reciprocal co-immunoprecipitation of protein complexes in yeast microsomes.

When full length His-tagged PtrC4H1 and untagged PtrC3H3 were co-expressed in yeast and lysates incubated with anti-His antibody, precipitation of PtrC4H1 was highly enriched in PtrC3H3 (Chen et al., 2011). The results were supported by reciprocal tagging followed by affinity purification and quantitative MS (spectral counting). Further experiments using BiFC, chemical crosslinking, MS, and reciprocal immunoprecipitation in stem differentiating xylem extracts provide a strong evidence for a multi-protein complex of PtrC4H1, PtrC4H2, and PtrC3H3 in yeast microsomes, differentiating xylem protoplasts, and in the differentiating xylem of the growing plant (Chen et al., 2011). The evidence for a trimeric protein complex suggests a structure for metabolic channeling; however, the complex was able to convert exogenous *p*-coumarate into caffeic acid, which argues against channeling.

### Interaction of Monolignol Enzymes With Other Pathway Proteins

**Figure 2:**
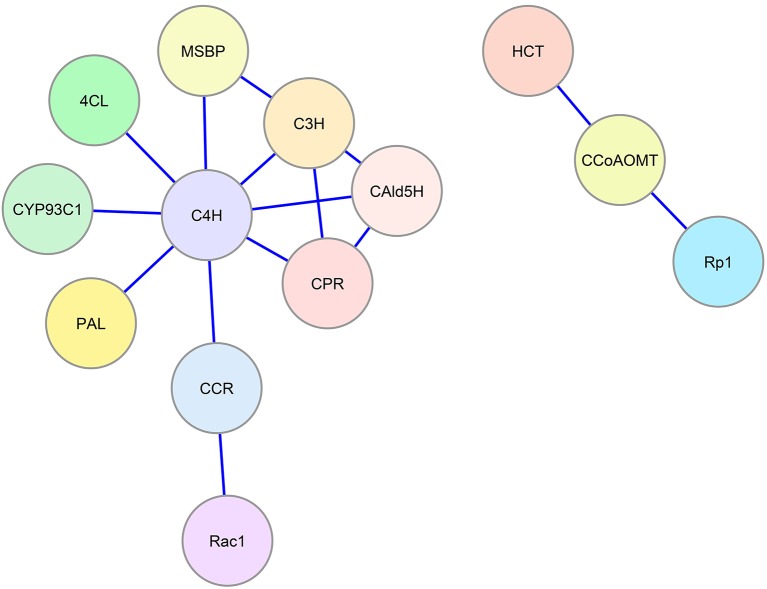
Enzyme-enzyme interaction network for the monolignol biosynthetic pathway. Blue lines represent protein-protein interactions indicated in the literature that involve monolignol biosynthetic enzymes. Colored circles represent the interacting enzyme families (refer to Figure 1).

Protein interactions involving monolignol biosynthetic enzymes have been associated with plant defense signaling. Rice *Oryza sativa* CCR1 interacts with a Rac family small GTPase (Rac1) in yeast and *in vitro* in a GTP-dependent manner (Kawasaki et al., 2006). Rac1 is a signaling protein that regulates the production of reactive oxygen species mediated by NADPH oxidase and has an important role in defense response. The interaction of Rac1 with CCR1 (Figure 2) leads to the enzymatic activation of CCR1 *in vitro* and in rice suspension cell cultures, which results in a higher accumulation of lignin (Kawasaki et al., 2006). Maize CCoAOMT and HCT interact with a plant disease resistance (R) protein, Rp1, which is a nucleotide binding Leu-rich-repeat (NLR) protein that confers pathogen resistance (Wang and Balint-Kurti, 2016). Physical interaction among CCoAOMT, HCT, and Rp1 in a multi-protein complex (Figure 2) suppresses the hypersensitive response to *Agrobacterium tumefaciens* infection conferred by Rp1. Downregulation of CCoAOMT or HCT in tobacco *N. benthamiana* disrupts the protein complex and re-activates Rp1, leading to a severe hypersensitive response to the infection (Wang and Balint-Kurti, 2016).

Recent work in Arabidopsis (Gou et al., 2018) has implicated two membrane steroid binding proteins (MSBP1 and MSBP2) in the structural organization of the three lignin P450 hydroxylases. These MSBPs were shown to reside on the ER membrane and to interact with C4H, C3H, and F5H forming MSBP-P450 complexes ( Figure 2). The MSBPs are proposed to be essential for the stability and activity of the P450s and necessary for channeling of metabolic flux through monolignol biosynthesis (Gou et al., 2018). In Arabidopsis, two cytochrome P450 reductases (ATR1 and ATR2) were shown to interact with the P450 enzymes (Sundin et al., 2014). One of these, ATR2, is associated with lignin biosynthesis and other phenylpropanoid biosynthetic genes. Plants with loss of function mutations in ATR2 were slightly reduced (6%) in total lignin, were enriched in *p*-coumaric acid derivatives, and were reduced in coniferyl alcohol derivatives. The results were attributed to reduced AtC3H and AtF5H activities (Sundin et al., 2014). Using a yeast-2 hybrid (Y2H) screen, strong interactions with ATR2 were found for AtC4H and AtC3H. Y2H evidence was also obtained indicating direct interactions of AtC4H and AtC3H with At4CL-1 as well as AtC4H with AtCCR1 (Gou et al., 2018). Affinity chromatography and co-purification of the three cytochrome P450 enzymes supported an *in vivo* association of these enzymes. All three P450s were found to interact with the MSBPs presumably on the ER membrane. BiFC experiments show an association of the P450s with MSBPs on the ER. Simultaneous downregulation of both MSBP genes reduced lignin biosynthesis (Gou et al., 2018).

### Outlook

A fundamental challenge of biochemistry is the reconstruction of an *in vitro* system, which 1) reproduces *in vivo* function, 2) is consistent with predictions of mathematical models, and 3) accounts for variation in place and time, as well as concentration of enzymes and metabolites (Mendes et al., 1995; Faraji et al., 2018). We are still far from being able to recreate such *in vivo* systems. One long-term strategy would be to build models based on *in vitro* evidence and to make predictions that can be verified in transgenic plants. Reconstruction of the pathway *in vitro* could also provide the necessary evidence. At this time, models of lignin biosynthesis have defined most, but most likely not all, of the components of the system for biosynthesis of monolignols. The best models have incorporated the absolute concentrations of the enzymes and metabolites, including their roles as substrates and inhibitors (Wang Jack et al., 2016; Wang et al., 2018). The variation in cell types and the relative abundance of transcripts of all known monolignol biosynthetic genes have been described (Shi et al., 2017). However, the current models are obtained from data where all enzymes are derived from a single wood-forming tissue (containing fiber, vessel, and ray cells) at the same place and stage of development. The models assumed enzyme functions to be largely independent (Wang et al., 2014; Wang et al., 2018). Cell type–specific data have not yet been incorporated, except to show that proteins that interact *in vitro* are co-expressed in the same cell types (Shi et al., 2017; Wang et al., 2018). Discovery of new regulatory components, such as the recently identified heterodimeric interaction between CAD and CCR that activates the sequential reduction of phenolic CoA esters to aldehydes and monolignols (Figure 1) (Yan et al., 2018), will continue to improve our understanding of the pathway. Incorporating the interactions of enzymes (Figure 2) and their metabolites requires a higher level of mechanistic insight based on dynamic interactomic models. The experimental basis for such models is available using inducible promoters that allow induction and repression of gene-specific expression (Borghi, 2010).

## Author Contributions

All authors listed have made a substantial, direct and intellectual contribution to the work, and approved it for publication.

### Conflict of Interest Statement

The authors declare that the research was conducted in the absence of any commercial or financial relationships that could be construed as a potential conflict of interest.

The handling editor is currently organizing a Research Topic with one of the authors RS, and confirms the absence of any other collaboration.
